# Gut Microbiota Metabolites: Unveiling Their Role in Inflammatory Bowel Diseases and Fibrosis

**DOI:** 10.3390/ph17030347

**Published:** 2024-03-07

**Authors:** Francesca Bernardi, Ferdinando D’Amico, Sarah Bencardino, Ilaria Faggiani, Jacopo Fanizza, Alessandra Zilli, Tommaso Lorenzo Parigi, Mariangela Allocca, Silvio Danese, Federica Furfaro

**Affiliations:** Gastroenterology and Endoscopy, IRCCS Ospedale San Raffaele, Vita-Salute San Raffaele University, 20132 Milan, Italy; bernardi.francesca@hsr.it (F.B.); damico.ferdinando@hsr.it (F.D.); bencardino.sarah@hsr.it (S.B.); faggiani.ilaria@hsr.it (I.F.); fanizza.jacopo@hsr.it (J.F.); zilli.alessandra@hsr.it (A.Z.); parigi.tommaso@hsr.it (T.L.P.); allocca.mariangela@hsr.it (M.A.); danese.silvio@hsr.it (S.D.)

**Keywords:** inflammatory bowel disease, Crohn’s disease, ulcerative colitis, microbiota, fibrosis, tryptophan, bile acids, short-chain fatty acids

## Abstract

In recent years, there has been a growing focus on the intricate interplay between the gut microbiota and host health, specifically in the context of inflammatory bowel diseases (IBDs). The gut microbiota produces a diverse array of metabolites, influencing the host’s immune response and tissue homeostasis. Noteworthy metabolites, such as short-chain fatty acids, bile acids, and indoles, exert significant effects on intestinal inflammation and fibrosis. This review integrates current research findings to clarify the mechanisms through which gut microbiota metabolites contribute to the progression of IBD and fibrosis, offering insights into potential therapeutic targets and strategies for managing these intricate gastrointestinal conditions. The unraveling of the complex relationship between gut microbiota metabolites and inflammatory processes holds promise for the development of targeted interventions that could lead to more effective and personalized treatment approaches for individuals affected by IBD and subsequent intestinal fibrosis.

## 1. Introduction

Inflammatory bowel disease (IBD) refers to chronic and recurrent inflammatory conditions that adversely affect the gastrointestinal tract [1]. While IBDs have been predominantly prevalent in Western countries, their incidence is swiftly rising, and their prevalence in Asia is contributing to their emergence as a global epidemic impacting both developed and developing nations [1,2,3].

IBDs are commonly linked to dietary habits, genetic predisposition, and the interplay of abnormal immune responses and environmental factors. However the precise pathogenesis remains elusive [4,5]. In recent times, the gut microbiota has emerged as a crucial environmental factor in the development of IBD [6]. The human intestinal tract is inhabited by approximately 160 significant bacteria out of the 1000 to 1150 bacterial species present [7]. In individuals with IBD, the biodiversity of the mucosa-associated microbiota and feces is reduced [8]. Proteobacteria and Actinobacteria phyla are abundant in IBD patients, while Firmicutes and Bacteroidetes, the main phyla of the healthy human gut microbiome, are depleted [2].

Moreover, the microbiome that resides in the cecum and colon has the ability to generate undigested dietary fiber, proteins, and peptides. It can synthesize, modulate, and break down numerous bioactive metabolites, some of which serve as crucial signaling molecules that play a role in promoting human health within the gut and other organs [9]. Changes in the microbial composition can lead to modifications in the bacterial metabolome, a product of gut microbial activities. Some metabolites, such as short-chain fatty acids (SCFAs), tryptophan, bile acids, and various small molecules, play crucial roles in IBD by influencing both intestinal permeability and the immune response [9,10]. Particularly, the host immune system can detect gut bacterial metabolites beyond pathogen-associated molecular patterns [10]. The recognition of these small molecules significantly influences not only the host immune response but also disease and inflammation in the gut [10]. Furthermore, bacteria can influence barrier function by controlling apoptosis among intestinal epithelial cells, producing essential proteins for tight junctions, or impacting the mucus layer [11].

The gut microbiota might also contribute to the development of fibrosis, which is an evolution of mucosal inflammation, leading to complications in individuals with IBD [12]. Fibrosis is characterized by an overabundance of extracellular matrix (ECM) accumulation, which ultimately results in organ failure due to chronic tissue injury, poor wound healing, and an underlying proliferation of mesenchymal cells [13]. These processes take place in both types of IBDs but manifest in specific locations: in ulcerative colitis (UC), the colonic mucosa and submucosa are impacted, whereas in Crohn’s disease (CD), fibrosis involves the entire thickness of the small or large bowel wall, including the muscularis propria and serosa [14]. Fibrosis is generated by an exaggerated response mediated by mesenchymal cells (fibroblasts, myofibroblasts, and smooth muscle cells) and the ECM in response to inflammatory damage [15]. Activation of mesenchymal cells can occur via autocrine factors, paracrine signals, microbe-associated molecular patterns, damage-associated molecular patterns, or the binding of other pattern recognition receptors [12]. While immunosuppressants, biologics, and small molecules can manage intestinal inflammation [16], there are currently no targeted therapies specifically designed to address fibrosis. Although recent preliminary findings suggest that the early and extended utilization of immunosuppressive or biologic agents might reduce the need for surgery and hospitalizations [17], it remains imperative to comprehensively grasp the mechanisms that underlie intestinal fibrosis. Such understanding is pivotal for the advancement of preventive and therapeutic approaches.

The main aim of this work is to summarize the data available on the relationship between microbiota and IBD with a particular focus on the mechanisms responsible for intestinal fibrosis.

## 2. Microbiota Metabolites

### 2.1. Short-Chain Fatty Acids (SCFAs)

SCFAs are a group of fatty acid compounds with an alkyl chain shorter than six carbons that includes butyrate, acetate, and propionate [18]. They are products of microbial fermentation of mainly undigested dietary fiber [18,19,20,21]. SCFAs are found in both small and large intestines, except for butyrate, which is mostly located in the colon and cecum [22]. In passing through the intestinal epithelium, SCFAs interact with host cells, influencing the immune response [23]. Their positive effects range from strengthening the intestinal barrier [24] and supplying ample energy to the gut epithelial cells and to the microbiota [25]. Additionally, they exert various functions on the physiology and immunity of the host, being considered metabolites with significant anti-inflammatory properties [26,27].

The composition of the intestinal microbiota and the intake of dietary fibers influence the concentration of SCFAs in the colon [28]. A fiber-rich diet may promote the presence of bacteria capable of hydrolyzing cellulose and xylan, such as those belonging to the genera *Prevotella*, *Xylanibacter*, and *Faecalibacterium prausnitzii* (a butyrate producer) [19,27]. It has been hypothesized that the abundance of F. prausnitzii and other SCFA-producing bacteria may protect the host from inflammation and non-infectious diseases of the colon [18]. The deficiency of *F. prausnitzii* is found in patients with Crohn’s disease [28,29].

SCFAs are inhibitors of histone deacetylase, promoting a tolerogenic and anti-inflammatory cellular phenotype essential for maintaining immunological homeostasis [30]. An example of how the microbiota influences the immune system through epigenetic mechanisms is demonstrated by the action of butyrate on the regulation of Treg cell differentiation: when naive CD4+ T cells undergo differentiation into Tregs in the presence of butyrate, there is a notable augmentation in histone H3 acetylation on lysine 27 (H3K27) at the Foxp3 promoter, as well as activators of CNS1 and CNS3 [31]. These epigenetic alterations amplify Foxp3 expression and, consequently, booster the regulatory capacity of Tregs [31].

SCFAs also impact defense mechanisms by enhancing the barrier function of the intestinal epithelium, inducing the differentiation of goblet cells, promoting mucin production, and facilitating the assembly of tight junctions [32,33,34]. SCFAs support epithelial homeostasis by inducing the production of IL-18 through the activation of inflammasomes [35]. Colonization by Bifidobacterium longum, which produces high levels of acetate, provides protection against lethal infection by *Escherichia coli* O157:H7 [36]. This suggests that SCFAs strengthen the integrity of the epithelial barrier, inhibiting the translocation of lethal toxins from the lumen to the systemic circulation [36] (Table 1).

#### SCFAs and IBD

The dysbiosis commonly observed in IBD is linked to a reduction in bacterial species that generate butyrate, including *Faecalibacterium prausnitzii* [37] and *Roseburia hominis* [38].

In addition, studies found a decrease in fecal SCFA levels in individuals with IBD [39,40,41], aligning with findings from quantitative PCR targeting the butyryl CoA:acetate CoA-transferase gene (primary mechanism for butyrate synthesis in the human microbiome) [42,43]. In contrast, lactate, the intermediate molecule, has been found to be elevated in instances of active UC and CD [44,45,46,47].

In murine models, research has unveiled that the lack of the fatty acid receptor GPR43 leads to non-responsive colitis [48]. In mice co-housing experiments, antibiotic treatments and evaluations of fecal butyrate levels demonstrated that excessive activation of the NLRP1A receptor results in a reduction in butyrate-producing Clostridiales, coupled with an increased production of IL-18 and interferon gamma (IFNγ) [49]. Particularly noteworthy is the identification of increased NLRP1 gene expression in inflamed regions of the distal colon in individuals with UC [49]. Additionally, a significant portion of bacteria exhibiting an inverse correlation with NLRP1, IL18, and IFNγ expression belonged to the order Clostridiales, thus establishing a mechanistic link in a human cohort [49].

The decreased level of SCFAs and SCFA-producing bacteria, given their role in regulating the differentiation of Treg and Teff cells, leads to a dysregulation in the balance between these cell types and to an increased production of proinflammatory cytokines [50,51]. The disturbances lead to mucosal layer damage, resulting in a compromised barrier function [11]. This facilitates bacterial infiltration, triggering an inflammatory cascade against the invading bacteria [52]. Additionally, pathobionts, as Proteobacteria, normally harmless bacteria that can exhibit pathogenic traits under specific conditions, thrive and proliferate in the inflamed environment [52]. This perpetuates inflammatory conditions, enabling adapted pathobionts to persist and further inhibiting the growth of commensal bacteria [52,53]. The depletion of commensals, emergence of pathobionts, and disruption in immune regulation culminate in a chronic inflammatory state [53].

**Table 1:** Roles of SCFAs.

SCFAs: Beneficial Effects	SCFAs: Reduction in IBD
− Strengthening the intestinal barrier [24] − Supplying energy to gut epithelial cells [25] and microbiota − Suppressing inflammation [26]	− Production of proinflammatory cytokines [50,51] − Compromised mucosal barrier function [11] − Pathobionts infiltration and loss of commensals [52,53]

### 2.2. Bile Acids

Primary bile acids, such as cholic acid (CA) and chenodeoxycholic acid (CDCA), are small molecules derived from cholesterol synthesis in the liver and are conjugated to either taurine or glycine [54]. The enzymatic activity of 7α/β-dehydroxylation enzymes facilitates the conversion of primary bile acids to secondary bile acids as deoxycholic acid (DCA) and lithocholic acid (LCA) [54]. The majority of these bile acids re-enter the enterohepatic circulation by being absorbed in the distal ileum [54]. Bile acids play a crucial role in lipid digestion and absorption within the small intestine [55]. In addition to governing their own synthesis, bile acids play a pivotal role in various metabolic, homeostatic, and immune functions by engaging with the farnesoid X receptor (FXR), transmembrane G protein-coupled receptor 5 (TGR5), pregnane X receptor, vitamin D receptor, and constitutive androstane receptor [56]. TGR5 contributes to enhanced insulin sensitivity (via GLP1) and increased energy expenditure in muscle and brown adipose tissue, inducing a reduction in lipogenesis and suppression of hepatic gluconeogenesis [56,57]. Furthermore, evidence indicates that the binding of bile acids to TGR5, situated within the intestinal stem cell reservoir, triggers the activation of the SRC/yes-associated protein (YAP) pathway [58]. This axis effectively governs intestinal stem cell maintenance, homeostatic renewal, and injury-induced regeneration [58]. FXR mitigates the response of Kupffer cells to lipopolysaccharide through the inhibition of nuclear factor-κB, consequently diminishing the release of IL-1, IL-6, and TNF from peripheral blood monocytes in humans, promoting liver regeneration and the production of antimicrobial peptides [59,60,61]. The activation of FXR by bile acids, particularly DCA, elucidates a significant role in intestinal wound healing: it facilitates the regeneration of intestinal crypts by inhibiting the cytosolic phospholipase A2 (cPLA2) enzyme, which is essential for the synthesis of prostaglandin E2 (PGE2) [62].

Bile acids and the microbiota reciprocally influence one another [63]. The deconjugation of amino acid residues from primary bile acids, facilitated by bile salt hydrolases, is a shared characteristic across various archaeal and bacterial divisions [64], for example, bile-salt-hydrolase-expressing Escherichia coli [65]. Additionally, the conversion of primary bile acids to secondary bile acids occurs within the colon, a process confined to a specific subset of clostridial species and facilitated by 7α/β-dehydroxylation enzymes [63]. Bile acids exert a significant impact on the composition and density of the gut microbiota: activation of FXR in the small intestine hinders bacterial overgrowth and translocation [66,67]. Bile acids exhibit both direct antimicrobial effects, exemplified by cholic acid (CA) and deoxycholic acid (DCA) on Bifidobacterium breve and Lactobacillus salivarius [68], and indirect effects, including the stimulation of host production of antimicrobial peptides such as cathelicidin [69,70], angiogenin I [66], and inducible nitric oxide synthase [71]. CA could induce an increase in Clostridia and subclass Erysipelotrichi while reducing members of the phyla Bacteroidetes and Actinobacteria [72]. Furthermore, secondary bile acids, as DCA, play a pivotal role in promoting colonization resistance to Clostridioides difficile infection [73] (Table 2).

#### Bile Acids in IBD

In IBD patients, gut dysbiosis exerts notable effects on metabolic processes, in particular reducing the processes of deconjugation and 7α-dehydroxylation, resulting in a decrease in secondary bile acids, including DCA, LCA, and tauro-LCA, and a concomitant increase in primary and conjugated bile acids, such as CA, CDCA, and G/TCA [44]. Also, dysbiosis involves an increase in Proteobacteria and Fusobacteria and a decrease in various phyla, notably Firmicutes, including Clostridiales [74]. Ileitis is identified as an important factor behind the absolute elevations of primary bile acids and, consequently, alteration in microbial diversity with a reduction in F. prausnitzii and its produced acetate-and l-methionine enzyme (MetY enzyme) [75,76]. On the contrary, Battat et al. illustrated that ileocolectomy in Crohn’s disease, while reducing the absorption of both bile acids, does not alter the capacity to convert primary bile acids to secondary bile acids; therefore, their metabolism does not exert an impact on ileal inflammation [76].

The expression and activity of bile-acid-activated receptors such as the farnesoid X receptor (FXR) and G-protein bile-acid-activated receptor (GPBAR1) are strongly influenced by the composition of the intestinal microbiota, and their functionality is adversely affected by intestinal inflammation [77]. Mice that lack FXR (FXR−/−) are more susceptible to chemical injury, such as patients diagnosed with CD, where the expression of FXR was reduced in biopsy samples [71,77]. Similarly, mice lacking GPBAR1 exhibit significant inflammation and have a deficiency in both Treg cells and M2 macrophages, mainly due to decreased IL-10 function and the inability to produce a counter-regulatory response during inflammation [78]. These factors increase intestinal inflammation, which could lead to developing or relapsing an IBD [78].

**Table 2:** Roles of bile acids.

Bile Acids: Beneficial Effects	Bile Acids in IBD: ↓ Secondary BA, ↑ Primary BA
− Lipid digestion and absorption [55] − ↓ lipogenesis and hepatic gluconeogenesis [56,57] − Liver regeneration [59,60,61] − Production of antimicrobial peptides [59,60,61] − Intestinal barrel homeostasis and regeneration [58] − Intestinal wound healing [62]	− Bacterial overgrowth and translocation [66,67] − Increasing inflammation (↓ IL-10, Treg, M2) and inability to produce a regulatory response during inflammation [78]

### 2.3. Tryptophan

Tryptophan is an essential, aromatic amino acid acquired through the diet by humans; it is found in dairy, poultry, fish, and oats [79]. Once eaten, tryptophan could follow kynurenine or serotonin pathways (host pathways) from which originate neuroactive compounds such as serotonin, melatonin, nicotinamide, and others [79,80,81]. One more pathway, operated by host microbiota, is the indole pathway, from which originate indole metabolites such as indoleacetic acid, indole-3-acetaldehyde, indole-3-aldehyde, indoleacrylic acid (IA), and IPA (that preserves the barrier function and suppresses mucosal TNF production) [82]. They are ligands and agonists of the aryl hydrocarbon receptor (AhR), a transcription factor with important anti-inflammatory roles: it regulates the T cell immunity by tissue-dependent influences and the innate lymphoid cells [82], and plays a role in the production of IL-22 [83]. AhR shows an important role in IBD pathogenesis [84]: it regulates the differentiation and function of T cells, and its expression is reduced in individuals with IBD [85].

AhR plays a crucial role in maintaining the homeostasis of the intestinal barrier: A reduction in or deletion of AhR in intestinal epithelial cells could lead to a compromised barrier and uncontrolled proliferation of intestinal stem cells, ultimately leading to malignant transformation [86]. Specifically, AhR regulates negative transcriptional regulators of the Wnt-β-catenin pathway, such as Rnf43 and Znrf3, which are E3 ubiquitin ligases, restricting intestinal steam cells proliferation and serving as a protective mechanism against tumorigenesis [86].

Diet-derived AhR agonists influence the preservation of microbial abundance, composition, and immune tolerance mediated by intraepithelial lymphocytes in the proximal small intestine [87], while microbiota-derived AhR agonists, produced mostly by *Peptostreptococcus russellii* and members of *Lactobacillus*, perform in the distal small intestine and colon [88].

Additionally, microbiota and its metabolites have an important regulatory role in kynurenine or serotonin pathways [89,90] (Table 3).

#### Tryptophan in IBD

Nikolaus S et al. demonstrated that tryptophan deficiency can contribute to the development of IBD or aggravate disease activity in a cohort of 535 patients [91]. Further evidence supporting this hypothesis includes a decrease in the kynurenine pathway in these patients [92], diminished AhR expression in inflamed mucosal samples obtained from individuals with CD [85], and a deficiency in dietary tryptophan linked to exacerbated colitis in murine models [93].

Several research studies conducted on mouse models have demonstrated the beneficial role of AhR [92]. Mice with knockout for caspase recruitment domain-containing protein 9 (Card9) and DSS-induced colitis showed reduced levels of indole derivative indoleacetic acid and a decreased ability of microbiota to activate AhR [92]. The same effect was observed in mice with knockout for IL-22: colitis was healed with the administration of IL-22 [92]. In fact, studies have shown that the increased level of IL-22 induced by AhR agonists, along with the reduction in IFNγ in mononuclear cells of lamina propria, determines healing from chemical and T cell transfer-model-induced colitis [85]. This effect is lost in blocking IL-22 activity [85]. In addition, AhR-activating *Lactobacillus* has been demonstrated to decrease the severity of colitis [92,94].

Other commensals producing the AhR agonist by the same pathway are *P. russellii*, a mucin-utilizing bacteria that metabolizes tryptophan to IA, and *C. sporogenes*, a producer of IPA [95]. Both bacteria present an fldAIBC phenyllactate gene cluster, which is found to be decreased in patients with UC [95]. P. russellii reduces the risk of colitis by enhancing the differentiation of goblet cells and inhibiting inflammatory pathways [95].

Karakan T et al. demonstrated that serum tryptophan, kynurenine, and picolinic acid values exhibited statistically significant reductions in patients during the active phase compared to those in remission (*p* = 0.01, *p* < 0.001, *p* = 0.022, respectively) [96], another proof of the anti-inflammatory effects of these molecules.

**Table 3:** Roles of tryptophan.

Tryptophan: Beneficial Effects	Tryptophan: Reduction in IBD
− Activating AhR and its anti-inflammatory and anti-tumorigenesis roles [82,83] − Suppressing mucosal TNF production [97] − Preservation of microbial abundance [87,88]	Development of or aggravating disease activity by: − Decreasing kynurenine pathway [92] − Diminishing AhR expression [85]

## 3. Microbiota-Induced Fibrosis

Fibrogenesis represents a physiological response activated by inflammation, with the potential outcomes of either tissue repair or fibrosis determined by the equilibrium between the generation and breakdown of extracellular matrix (ECM) proteins [98]. It serves as the ultimate pathological consequence in the majority of chronic inflammatory conditions and significantly contributes to the dysfunction and failure of organs [99]. Despite the growing acknowledgment of fibrosis as a concern, there are scarce or virtually no available treatment strategies at present [100].

While fibrosis is a prevalent issue in IBD, the factors that initiate chronicity and foster fibrosis remain unknown [101]. In UC, the accumulation of the extracellular matrix (ECM) is confined to the mucosal and submucosal layers of the colon, resulting in the shortening and stiffening of the colon [100]. CD commonly presents intestinal fibrosis as a complication, resulting in bowel wall thickening and strictures [102]. There is notable diversity in intestinal fibrosis among individuals with IBD, indicating a potential genetic component to fibrosis susceptibility that is influenced by environmental and intestinal microbial factors [100]. Researchers observed a significant rise in collagen accumulation in specific regions of the colonic walls in germ-free mice following the inoculation of a fecal suspension obtained from healthy specific-pathogen-free (SPF) rats [103]. Furthermore, individuals with CD exhibit circulating antibodies against microbial antigens derived from Saccharomyces cerevisiae or Pseudomonas fluorescens, associated with clinical features of intestinal fibrotic stenosis and surgical interventions, suggesting that the gut microbiota can contribute to fibrosis both directly and indirectly [104].

**Figure 1:**
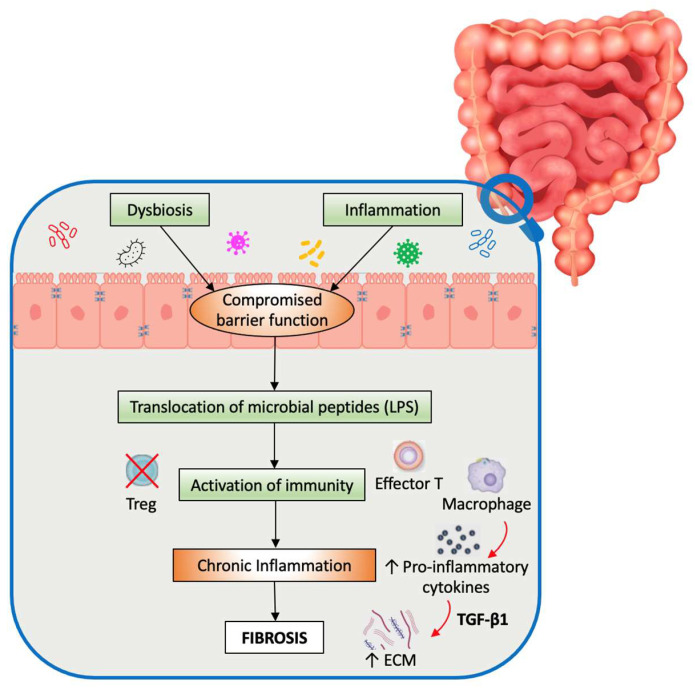
Pathogenesis of fibrosis in IBD. Dysbiosis and inflammation compromise the integrity of the intestinal epithelial barrier. Gut microbes are consistently exposed within intestinal immune and non-immune cells. This exposure initiates intracellular signaling, leading to the production of pro-inflammatory cytokines, including TGF-β, and fostering chronic inflammation. The resultant outcome is the deposition of extracellular matrix (ECM), culminating in intestinal fibrosis.

When dysbiosis and inflammation compromise the integrity of the intestinal epithelial barrier, gut microbes are consistently exposed to intestinal immune and nonimmune cells, initiating intracellular signaling via their pattern recognition receptors (PRRs), like Toll-like receptors (TLRs) and Nod-like receptors (NLRs), which recognize pathogen-associated molecular patterns (PAMPs) and transmit intracellular signals (Figure 1) [12,16,105,106]. Lipopolysaccharide (LPS), a fibrogenic molecule found in the outer membranes of Gram-negative bacteria, interacts with Toll-like receptor 4 (TLR4) on the fibroblast membrane, which then oligomerizes and recruits downstream adaptors to its cytoplasmic toll-interleukin-1 receptor (TIR) domains [107]. The subsequent signaling event involves two pathways: MyD88-dependent and MyD88-independent [107]. In the MyD88-dependent pathway, TLR4 activation leads to the phosphorylation and degradation of inhibitory nuclear factor-kB (NF-kB) members, which results in the translocation of NF-kB to the nucleus, where it regulates gene transcription [107]. The regulation of gene transcription suppresses the expression of SMAD family member 7 (SMAD7). SMAD7 acts as a negative regulator of transforming growth factor beta 1 (TGF-β1) signaling. This suppression results in heightened TGF-β1 signaling and an increased secretion of ECM proteins [108]. Furthermore, when human fibrocytes encountered LPS, they showed increased collagen production compared to TGF-β1 exposure [109]. This suggests that LPS can increase fibrosis without depending on inflammatory stimulation of TGF-β1 [109]. Similar mechanisms involving peptidoglycan–polysaccharide, another bacterial cell wall polymer, could similarly increase TGF-β1 expression and enhance collagen accumulation in myofibroblasts [110].

Current research examines how particular microorganisms contribute to the progression of fibrosis in IBD patients [111,112,113]. In a mouse model of transgenic tumor necrosis factor-like cytokine 1A (TL1A) overproduction, *Mucispirillum schaedleri* and *Ruminococcus* in the cecum and *Streptococcus* and *Lactobacillus* in the ileum were positively linked with fibrosis in contrast to Oscillospira, Coprococcus, Faecalibacterium prausnitzii, and Bacteroides [111]. In animal models of IBD, adherent-invasive Escherichia coli (AIEC), a specific pathotype of *E. coli*, and *Salmonella enterica* serovar Typhimurium have been demonstrated to trigger inflammation by promoting an increase in T helper (TH) 1 and TH17 immune responses, with the subsequent development of fibrosis [112,113]. Furthermore, akin to the observations in patients with CD, mice infected with AIEC exhibited substantial ECM deposition, accompanied by elevated levels of collagen types I/III and increased expression of pro-fibrotic mediators, including transforming growth factor-1 (TGF-1), connective tissue growth factor, and insulin-like growth factor I (IGF-I) [113].

Genetic studies in humans provide evidence supporting the connection between the gut microbiota and the development of intestinal fibrosis [114]. Individuals harboring variations in the nucleotide-binding oligomerization domain 2 (NOD2) gene exhibit a higher susceptibility to CD [114,115]. Biologically, NOD2 serves as an intracellular PRR for muramyl dipeptide, derived from the peptidoglycan of both Gram-positive and Gram-negative bacteria [116]. Following intracellular stimulation by bacterial products, NOD2 initiates the NF-kB pathway, eliciting a defensive response to safeguard the host from bacterial infections [117]. Individuals with CD who possess a variant of the NOD2 gene face an elevated risk of experiencing complications and undergoing surgery [118]. A meta-analysis revealed that having at least one NOD2 variant raised the likelihood of stricture development in individuals with CD, while possessing two NOD2 mutations was associated with a 41% higher risk of complicated disease (stricturing or fistulizing subtype) and a 58% increased risk of requiring surgery [119]. These findings provide support for the concept that impaired bacterial sensing by NOD2 contributes to the initiation of intestinal fibrosis in CD [119]. Furthermore, there are reports indicating that the functional capacities of intestinal myofibroblasts differ between normal individuals and those with IBD, specifically CD [120]. Myofibroblasts obtained from individuals with CD exhibited a higher rate of proliferation compared to those from normal individuals and individuals with UC [120]. Additionally, the expression patterns of TGF-β isoforms varied in CD compared to normal or UC [117]. In CD myofibroblasts, there was a significant reduction in TGF-β3, while TGF-β2 was enhanced in comparison to normal or UC, suggesting that the distinct functional characteristics of myofibroblasts in CD may contribute to the development of intestinal fibrosis [120]. However, the role of the gut microbiota in regulating TGF-β isoforms remains unclear.

## 4. Treatment and Therapeutic Perspectives

The microbiota and its metabolites have a pivotal role in the development and pathogenesis of IBD. Consequently, by intervening in these mechanisms, specific prevention and therapeutic strategies have been assessed, which may serve as alternatives or complements to the conventional IBD therapy.

### 4.1. SCFAs

The production of butyrate by certain butyrate-producing bacteria is significantly diminished in patients with UC, leading to a reduction in SCFAs in the colonic lumen in UC [38]. A consortia-based therapy tested on germ-free mouse with inducted colitis is VE202 (Vedanta Biosciences in conjunction with Janssen Biotech), which includes 17 human-derived Clostridium strains, known to have butyrate-producing activity [121]. The study demonstrates that VE202 reversed histological colitis and other inflammatory end-points through a unique IL-10-independent protective mechanism, the correction of dysbiosis resulting in reduced levels of Enterobacteriaceae and Fusobacteria [122]. These findings could offer a basis and target for the therapeutic application of carefully chosen resident protective bacterial combinations in patients with IBD.

Further evidence comes from the administration of oral butyrate: it has the potential to enhance the effectiveness of oral mesalazine in treating active UC disease [20], and implementing a diet that elevates SCFA levels in individuals with IBD can also alleviate colitis [38].

A randomized, multicenter, double-blind, placebo-controlled, Phase 2b clinical trial investigated the safety and efficacy of SER-287, a naturally obtained blend of purified Firmicute spores, in inducing clinical remission after 10 weeks of an induction dose in mild-to-moderate UC in 203 patients [123]. Also, if, in a Phase 1b trial, administering SER-287 orally on a daily basis led to increased rates of clinical remission compared to the placebo (40% vs. 0%, respectively; *p* = 0.024) [123], no clinically (endoscopic improvement, endoscopic remission, or symptomatic remission) and statistical significant differences were noted in the absolute rates of clinical remission among the three treatment groups (10.3 percent for the full induction dose, n = 68, and 10.6 percent for the step-down induction dose, n = 66, compared to 11.6 percent for placebo, n = 69) [124]. Considering these findings, SER-301 was developed employing a computational optimization algorithm that incorporated the functional properties of individual strains with clinical data from SER-287 [125]. It showed, in vitro, to enhance the production of SCAFs, tryptophan, and bile acids and to decrease the secretion of IL-8 and IFN-gamma, acting as an anti-inflammatory drug [125]. SER-301 is presently undergoing assessment in a Phase 1b clinical trial including adults with active mild-to-moderate ulcerative colitis (ACTRN12620000963921) to examine the hypothesis that the engraftment of drug product species leads to a decrease in intestinal inflammation and the restoration of epithelial barrier damage [126].

### 4.2. Tryptophan

Indole compounds derived from plants have a history of use in traditional herbal medicine for treating IBD [127]. This historical use underscores the significance of the interaction between kynurenines and the aryl hydrocarbon receptor, and their impact on the immune system [80,127]. Simultaneously, Indigo Naturalis, a traditional Chinese medicine containing indirubin and indigo, which act as activators of AhR [128], exhibited favorable effects as a promising pharmaceutical candidate for the treatment of UC, as demonstrated in a study involving mice with dextran sulfate sodium (DSS)-induced UC [129,130].

Studies conducted on animals suggest that administering tryptophan or its metabolites may be a potential therapy for IBD [93,131,132,133]. Notably, the application of niacin or nicotinamide demonstrates a robust anti-inflammatory effect in animal models [132]. Tryptophan and its metabolites play a role in regulating gut microbiota homeostasis and could act as energy carriers for oxidative cellular processes in epithelial cells or cells of the mucosal immune system [93]. Nutritional supplementation with nicotinamide [93,132] or indole-3-aldehyde [133] has the potential to modify the microbiota, and high-dose administration may redirect tryptophan metabolism toward anti-inflammatory pathways [93,131,132,133].

The metabolism of tryptophan at the epithelial interface with the luminal microbiota could thus exert a significant regulatory influence on the inflammatory pathophysiology of IBD [127]. Consequently, tryptophan and its metabolites may not only serve as valuable biomarkers but could also represent a promising therapeutic target in IBD, particularly if administered through ileocolonic release formulations [133]. Prospective studies are essential to investigate the feasibility of manipulating tryptophan metabolism in IBD therapy.

Further, implementing a dietary intervention involving tryptophan can augment AhR activation capacity, presenting a potential therapeutic approach for individuals with IBD and associated intestinal dysbiosis [127].

### 4.3. Bile Acids

Several bile-acid-activated receptors exhibit alterations in individuals with IBD [134,135], leading to the dysregulation of bile acid signaling. This dysregulation mediates dysfunctional communication between the intestinal microbiota and the immune system in IBD patients [134,135]. Indeed, therapies based on bile acids could be explored for the treatment of IBD, and the restoration of bile acid signaling might prove beneficial in managing the bowel inflammation [136,137]. Considering the defective expression of farnesoid X receptor (FXR) in IBD, a therapeutic strategy could involve non-selective and intestinal FXR selective agents [71]. Another choice could be the use of GPBAR1 agonists: their expression is confined to the intestine, and evidence from preclinical models indicates that the GPBAR1 ligand has significant immune modulatory effects in rodent models of colitis [78]. Both these drugs, not yet available in formulations for IBD despite their potential utility, commonly exhibit itching as a side effect [137,138]. Nevertheless, UDCA, a modest GPBAR1 ligand, has been employed in the treatment of IBD, and experimental data indicate a potential beneficial role for this agent in this context [136,139,140]. Also, RORγt ligands show promise in the treatment of IBD, and efforts are underway to identify RORγt reverse agonists (antagonists) to address intestinal inflammation [141]. In addition, various strategies could be employed for the indirect modulation of intestinal FXR, GPBAR1, and RORγt by leveraging the intestinal microbiota through the utilization of probiotics, prebiotics, or fecal microbial transplantation [142,143,144,145]. Strong scientific evidence is required to substantiate the effectiveness of these potential new treatments.

### 4.4. Fibrosis

Regarding the understanding of intestinal fibrosis, it has evolved from being considered as a static and irreversible condition to being recognized as a dynamic and reversible disease [101]. Current research is exploring innovative therapeutic approaches that target specific stages in fibrogenesis with the goal of decreasing or reversing fibrosis associated with IBD [146]. For example, anti-inflammatory and immunosuppressive drugs (anticytokines, anti-chemokines, and antigrowth factor blockers) could be able to modulate general fibrogenesis pathways [99]. Evidence also shows the role of the control of angiogenesis as a novel therapeutic approach by inhibiting profibrotic pathways [146]. Further, rectifying mucosal barrier permeability may reduce or eliminate the excessive absorption of bacterial products that activate immune and mesenchymal cells [147]. This approach could prove to be a viable strategy for the prevention or treatment of fibrosis.

Hence, it is crucial to investigate the correlation between fibrosis and the microbiota in IBD to identify potential pro-fibrotic or preventive microbial compositions. As previously discussed, the microbiota, through the metabolites that it produces, may be involved in the generation of pro-fibrotic cytokines such as TNF-α, IFN-γ, IL-6, and IL-17 [16,98,101]. Consequently, fibrosis could potentially be mitigated by modulating the microbiota, achieved through the use of probiotics, prebiotics, and fecal transplantation. By modifying the bacterial populations that inhabit the intestine, it might be possible to reduce the production of these cytokines implicated in fibrogenesis patterns and enhance the production of anti-inflammatory and antifibrotic cytokines, such as IL-10 [148,149]. Several in vitro and in vivo investigations have been conducted to evaluate the impact of probiotics and prebiotics on intestinal fibrosis (Table 4), but further studies are required to delve into this therapeutic hypothesis.

## 5. Conclusions

Advancements in gut microbiota research have elucidated that IBD is linked to an imbalance in the gut microbial community and its metabolites [53], which could influence intestinal permeability, immune response, and the development of fibrosis [117,149].

The therapeutic landscape for IBD is expanding beyond conventional strategies. By further investigation on this topic, our review highlights the importance of the role of microbiota and tries to summarize individualized and new therapeutic approaches, considering the heterogeneity of IBD manifestations and patient responses to treatment. Tryptophan, bile acids, and SCAFs offer promising avenues for targeted interventions, emphasizing the need for further research to translate these findings into clinically relevant treatments. The same applies to the development of intestinal fibrosis and its complications: the multifaceted nature of IBD requires a comprehensive understanding of these elements and their intricate interactions within the complex gut environment. Continued exploration of these pathways holds great potential for advancing precision medicine approaches in the management of IBD.

## Figures and Tables

**Table 4:** Studies about the therapeutic role of probiotics and prebiotics for intestinal fibrosis.

Agent	Mechanism	Model	Reference
Polyphosphate	↓ IL-1β, TNF-α	Cellular models: DSS- and TNBS-induced colitis	[150]
12 probiotics, prebiotics, rosavin and zinc	↓ IL-6, IL-1β, IL-17 ↑ IL-10	Mouse models: DSS-induced colitis	[151]
Lactococcus lactis ML2018	↓ NF-κB and MAPK signaling ↑ SCFAs	Mouse models: DSS-induced colitis	[152]
4 probiotics	↓ IL-1β, TNF-α ↓ TLR4/NF-κB and TGF-β1/Smad signaling ↑ microbial balance	Rat models with abdominal adhesions	[153]
Multi-Strain Probiotic Formulation (Vivomixx)	↓ TGF-β1	Cellular models: CCD-18Co cells cultured with TGF-β1	[154]

↓: decreasing; ↑: increasing.

## Data Availability

No new data were created or analyzed in this study. Data sharing is not applicable to this article.
